# Therapeutic Potential and Challenges of Mesenchymal Stem Cell-Derived Exosomes for Peripheral Nerve Regeneration: A Systematic Review

**DOI:** 10.3390/ijms25126489

**Published:** 2024-06-12

**Authors:** Clelia Dogny, Dominik André-Lévigne, Daniel F. Kalbermatten, Srinivas Madduri

**Affiliations:** 1 Department of Plastic, Reconstructive and Aesthetic Surgery, Geneva University Hospitals, 1205 Geneva, Switzerland; 2 Bioengineering and Neuroregeneration Laboratory, Department of Surgery, University of Geneva, 1211 Geneva, Switzerland

**Keywords:** nerve repair, nerve regeneration, mesenchymal stem cells, exosomes, secretome

## Abstract

Gap injuries to the peripheral nervous system result in pain and loss of function, without any particularly effective therapeutic options. Within this context, mesenchymal stem cell (MSC)-derived exosomes have emerged as a potential therapeutic option. Thus, the focus of this study was to review currently available data on MSC-derived exosome-mounted scaffolds in peripheral nerve regeneration in order to identify the most promising scaffolds and exosome sources currently in the field of peripheral nerve regeneration. We conducted a systematic review following PRISMA 2020 guidelines. Exosome origins varied (adipose-derived MSCs, bone marrow MSCs, gingival MSC, induced pluripotent stem cells and a purified exosome product) similarly to the materials (Matrigel, alginate and silicone, acellular nerve graft [ANG], chitosan, chitin, hydrogel and fibrin glue). The compound muscle action potential (CMAP), sciatic functional index (SFI), gastrocnemius wet weight and histological analyses were used as main outcome measures. Overall, exosome-mounted scaffolds showed better regeneration than scaffolds alone. Functionally, both exosome-enriched chitin and ANG showed a significant improvement over time in the sciatica functional index, CMAP and wet weight. The best histological outcomes were found in the exosome-enriched ANG scaffold with a high increase in the axonal diameter and muscle cross-section area. Further studies are needed to confirm the efficacy of exosome-mounted scaffolds in peripheral nerve regeneration.

## 1. Introduction

Peripheral nerve damage is a significant health problem as it causes important morbidity among patients, such as loss of motor or sensory functions. Peripheral nerve regeneration is a complex process that involves several actors, including neurons, their axons, Schwann cells and inflammatory cells. From a clinical perspective, a crucial point is the length of the defect, as tension-free suture has to be achievable. Gaps smaller than 4 cm have a relatively good prognosis with regeneration rates of 1 mm/day, depending on the location of injury (proximal or distal) [1]. Classically, gaps greater than 4 cm cannot regenerate, and the gold standard method to repair those gaps is an autologous nerve graft transplant [2]. However, this technique is invasive and requires nerve harvesting at a secondary site, with a loss of sensation and esthetic consequences at the donor site, as well as a limited quantity of harvestable nerves. Another crucial factor is the type of nerve damage, with motor neurons showing more targeted, albeit incomplete, regeneration than sensory neurons [3,4].

Nerve repair following nerve injury is complex and includes axon degeneration at the distal stump and myelin degradation, known as Wallerian degeneration, followed by the arrangement of the Büngner bands that will guide the axonal regrowth from the proximal stump [5,6]. SCs secrete pro-inflammatory cytokines such as IL-1a and TNFα, recruiting macrophages, which are important in Wallerian degeneration [6]. The recruited macrophages degrade the residual myelin and have a major role in neovascularization, notably with the secretion of vascular endothelial growth factor (VEGF) [7]. Repair SCs also secrete many neurotrophic factors (nerve growth factor [NGF], brain-derived NF [BDNF], glial cell-derived NF [GDNF], neurotrophin-3 [NT3]) [8], which allows for axonal growth and survival. However, the repair phenotype and the survival of SCs at the distal stump are inversely proportional to the time it takes for the axon to regrow, which for large gaps can lead to a failure in regeneration [9,10].

SCs are peripheral glial cells and constitute one of the major actors in peripheral nerve regeneration. When trauma occurs, they change their phenotype and initiate a repair program, which in some aspects resembles that of immature SCs. The repair SCs will have several roles, such as initiating the inflammatory process, distal nerve degeneration, and maintaining and guiding axonal outgrowth to their target. At the end of the repair process, SCs shrink and myelinate the new axons [11]. Neurons also have a dynamic role in the regeneration of their axons and interact with other actors of nerve repair, including macrophages and SCs [12,13].

Many efforts have been made to develop alternatives to autologous nerve grafts using scaffolds to guide neuronal outgrowth. There is a wide variety of scaffolds, which are divided into non-degradable matrices, such as silicone, and degradable matrices, which include synthetic and natural polymers [14]. The ideal scaffold must be as close as possible to the intrinsic extracellular matrix of nerve tissue and thus ideally have the following characteristics, thus allowing the preservation or reformation of the peripheral nerve architecture and not acting as a barrier: no toxicity; no transmission of disease; good biocompatibility; and a good degradation rate [15]. It should also be economic and easy to manufacture, store and use.

Despite some promising results, biological matrices alone show limited regenerative potential, especially for longer defects, as they merely serve as a guide to direct axonal outgrowth. Efforts have been made to enhance the regenerative characteristics of scaffolds, including mounting scaffolds with growth factors SC, or mesenchymal stem cells (MSC) [16,17]. The effect of a variety of growth factors mounted on scaffolds has been investigated, ranging from a single factor, such as NGF, BDNF, NT3, ciliary neurotrophic factor (CNTF) and VEGF [18], to platelet concentrate of growth factors, which include platelet-derived GF (PDGF), VEGF, transforming GF (TGF) and insulin-like GF (IGF) [19,20]. However, growth factors are difficult to manipulate as their concentration and diffusion time need to be precisely tailored. They have a very short half-life and need modification to diffuse for a longer period [16,18]. Studies also tend to show that a single growth factor is not sufficient to ensure good regeneration [21,22].

The possibility of adding SCs directly into scaffolds to provide more support for nerve regeneration has been investigated [23,24]. However, SCs are difficult to cultivate in vitro and have a slow proliferation rate [24,25,26]. They also require the sacrifice of a healthy nerve, which does not make them optimal in the clinical setting. An option for not having to harvest or culture SCs that has been explored is the use of MSCs, which can differentiate into different lineages, and among these, the neurogenic lineage [27]. MSCs can be isolated from different niches, like the umbilical cord (umbilical cord-derived MSCs [UMSC]), the skin, bone marrow (bone marrow-derived MSCs [BMSC]), gingiva (gingiva-derived MSCs [GMSC]) and adipose tissue [28,29,30,31]. Adipose-derived SCs (ASCs) are particularly interesting in this respect as they can be easily harvested, e.g., with liposuction [32], which is a minimally invasive procedure.

The role of ASCs in peripheral nerve regeneration has been investigated in vitro and in vivo. In vitro, following differentiation protocols, ASCs can differentiate into Schwann cell-like cells (SCLCs) that have Schwann cell-like properties and display SC-specific markers such as S100, p75 and GFAP [33,34,35,36]. SCLCs have been shown, in vitro, to increase the number, length and density of neurites, and in vivo, to increase angiogenesis and axon growth, and sometimes to improve functional outcome [37].

However, there is a risk of de-differentiation once the substrate is removed [35]. Many studies have studied the potential effects of ASCs on peripheral nerve regeneration [38]. Some have noted an increase in myelination and fiber size and number, a better differentiation of SCs, and an improvement in nerve conduction and lower muscle atrophy. Other studies have shown that ASC transplantation was as effective as SC transplantation without the ASCs differentiating into SCLCs [39,40]. It is conceivable that the essential role of ASCs is to release growth factors that allow for the recruitment of local SCs and neurite outgrowth (BDNF, GDNF, CNTF, IGF.1, NGF, NT3, NT4) [31,36,41,42].

A crucial element for the efficacy of bioengineered scaffolds mounted with ASC is therefore their survival over time. However, the number of surviving ASCs after transplantation varies among studies, with some reporting a low survival rate at 14 days while others report about 5% [43,44], suggesting that there is an initial stimulation due to the ASCs, which would be more important than the differentiating potential of ASCs in SCs. As mentioned, SCs are particularly difficult to grow and multiply, and need to be harvested from a healthy nerve. They have an immunogenic potential if they come from an allogenic source. ASCs, although easier to obtain and multiply, still require an operation to harvest them, with associated risks. In rare cases only, ASCs have been reported to induce tumors in the tissue where they are injected [45,46].

**Figure 1:**
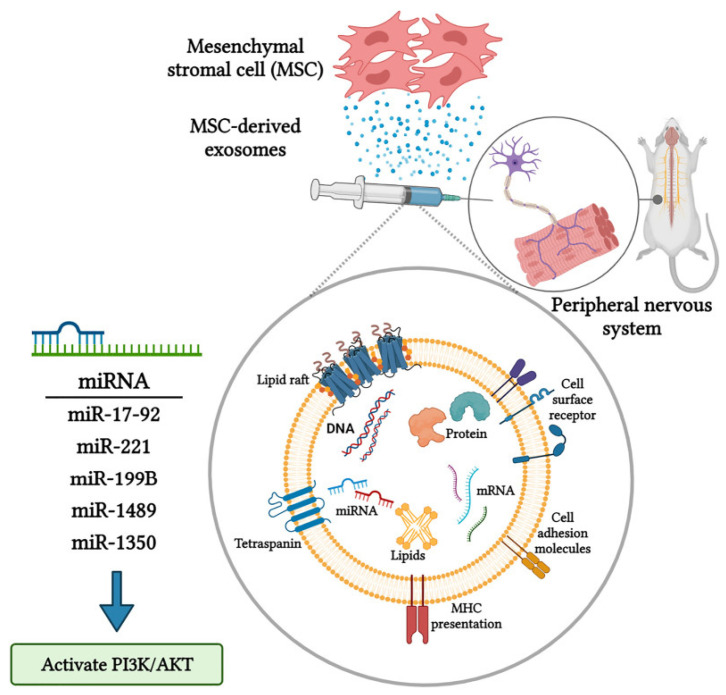
Molecular mechanisms regulating the axonal growth and nerve regeneration in response to the secretome derived from the mesenchymal stem cells. Image was reprinted from Namini Mojdeh et al., 2023 [52].

An extracellular communication pathway used by most cells, including ASCs, is that of exosomes, which are extracellular vesicles like apoptotic bodies and microvesicles. Exosomes, specifically, are nanovesicles (30–200 mm) with a lipid bilayer whose molecular content depends on the environment and the type of secreting cell, as they can emerge from almost all cell types [47]. Exosomes are easier to store and have a low immunogenicity compared to ASCs due to the few proteins on their surface and, therefore, a low rejection potential when used as an allograft [48,49]. This would allow to produce exosomes for patients on a large scale without having to isolate them from each patient individually, which would allow for the production of a ready-to-use scaffold. Exosomes exhibit specific markers, such as CD 81 and CD 63, as well as immunosuppressive molecules (PD-L1 and CD 200) and more specific signaling molecules, such as TFN and TGF-β [47]. The manufacturing process involves several phases, including early and late exosomes, which eventually fuse with the plasma membrane, releasing the exosomes into the extracellular space [50]. MSC exosomes have several roles. They can act on neurons by transmitting miRNA and growth factors such as GDNF, fibroblast GF (FGF-1), BDNF, IGF-1 and NGF (Figure 1). They also have an immunomodulatory role via the induction of IL10 and TGFβ [49]. Additionally, they are thought to play a role in angiogenesis [51].

**Figure 2:**
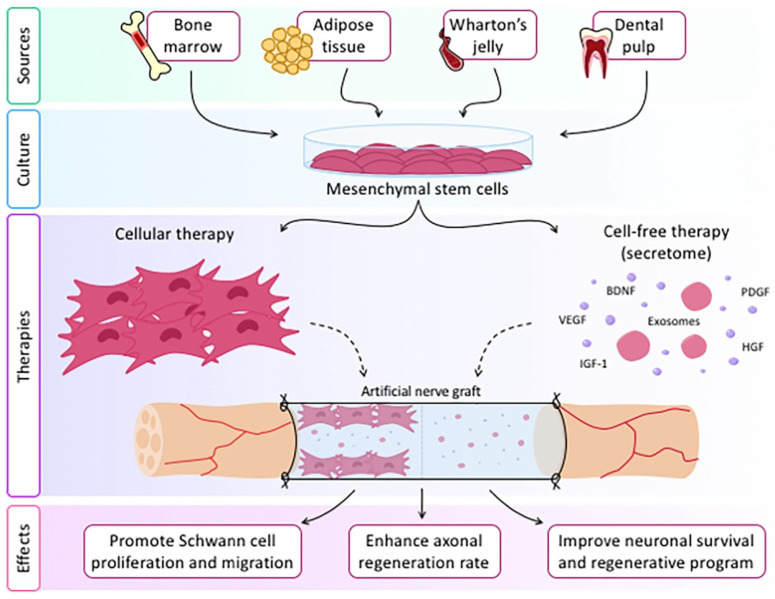
Applications of the nerve conduits loaded with secretome derived from different mesenchymal stem cells for the repair of the peripheral nerve injuries. Image was reprinted from Contreras Estefania et al., 2023 [57].

Studies have shown that secretome derived from different MSCs promote axonal growth in vitro and nerve regeneration in vivo [53,54] (Figure 2) and have been shown to promote SC proliferation and reduce their apoptosis [55]. An in vivo study also showed the non-inferiority of exosomes produced by undifferentiated ASCs compared to ASCs treated with an immunosuppressive molecule (FK506) that promotes nerve regeneration [56], which would be clinically valuable as there would be no need to transform ASCs. ASC-derived exosomes appear to offer interesting opportunities in the field of peripheral nerve regeneration treatment. Particularly at the clinical level, it would be interesting to have a ready-to-use system for the reconstruction of nerve gaps in an urgent clinical setting. This would require a system equal or superior to autologous nerve grafting and more clinically achievable than autologous ASC or SC harvesting and culture. The development of scaffolds mounted with ASC-derived exosomes provides a unique combination of potentially strong regenerative effects and a readily available off-the-shelf product with little immunogenicity. In this systematic review, we compare the different scaffolds available today for mounting ASC- and MSC-derived exosomes, with the following question, which are the most promising scaffolds and exosome sources currently in the field of peripheral nerve regeneration?

## 2. Materials and Methods

### 2.1. Search Strategy

This review was conducted according to PRISMA (Preferred Reporting Items for Systematic Reviews and Meta-Analysis) guidelines. PubMed, Embase and Google Scholar databases were used to search for studies, using the terms {peripheral nerve regeneration} and {exosomes}. The search yielded 81, 449 and 59 results, respectively. The review was conducted from May 2022 to September 2022 by a single author.

### 2.2. Inclusion and Exclusion Criteria

Our review was strictly limited to studies with a focus on peripheral nerve injury and which met the criteria of having scaffold and exosomes present. A first selection was independently performed by an initial screening of titles and abstracts, followed by a full-text review to confirm eligibility. Exclusion criteria were the following: reviews, meta-analyses, and articles not relevant to the topic and articles written in languages other than English. We have only included original research, which, based on the current state of research, had only been carried out on animal models.

## 3. Results

### 3.1. Systematic Review

**Figure 3:**
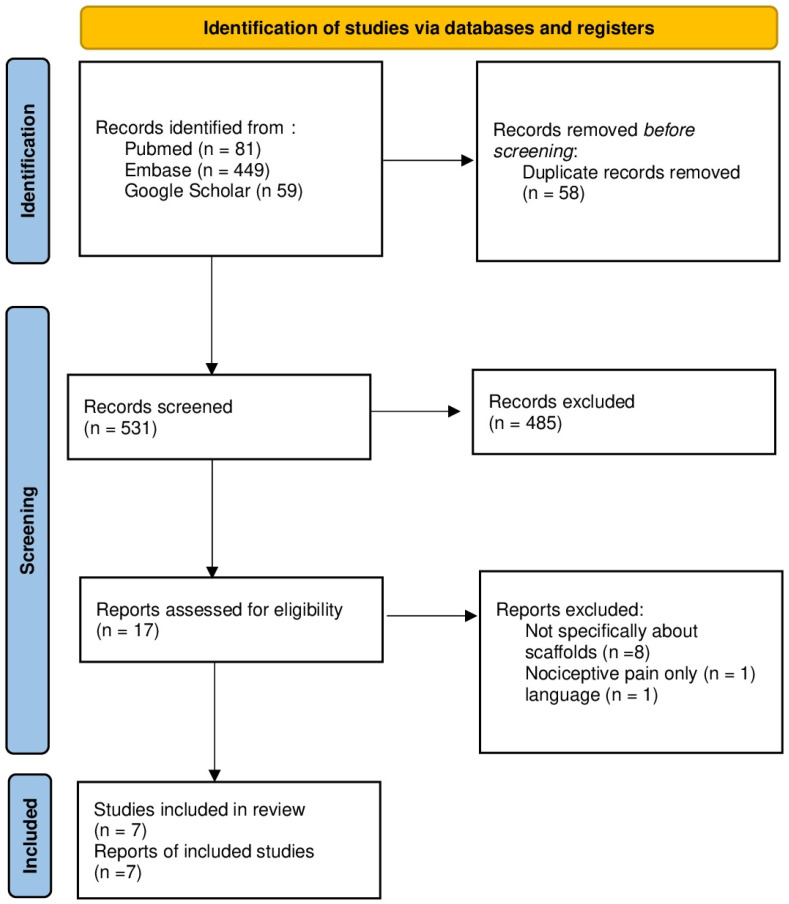
PRISMA flow chart.

A total of 589 articles were initially identified; 58 were duplicates. Of the 485 articles, 27 were reviews. Based on the title and abstract, a set of 17 articles were subjected to a full-text review, the other 441 were not relevant to this study. Eight were eliminated due to the lack of scaffolds, one was written in Chinese, and one focused on nociceptive pain. Seven fulfilled the criteria to be included in the review (Figure 3).

Of the seven articles selected, there was an important variety in the origin of the exosomes, matrices used, and the length of the gaps (Table 1). In two studies, exosomes were derived from ASCs [58,59]; otherwise, they were derived from UMSCs [60], BMSCs [61] and GMSCs [62]. Induced stem cells (iPCS) [63] and an exosome preparation [64] were only used in one article each. Most materials were degradable matrices, except for those used by Chen et al. and Yang et al., who used Matrigel and alginate in silicone [58,59]. Pan et al. and Ikumi et al. used acellular matrices [63,64], and Li et al. and Rao et al. used chitosan or chitin [61,62]. Liu et al. used hydrogel with two different densities [60]. Injury types ranged from a crush injury [60] to a 15 mm gap injury (Pan et al. [63]) with three articles using a 10 mm gap (Yang et al., Rao et al. al, and Ikumi et al. [59,62,64]). Li et al. used a 2 mm gap [61] and Chen et al. used a 7 mm gap [58]. The exosome concentration varied between 0.5 μg/μL and 1 μg/μL with final exosome concentrations varying drastically between 10 μg [62] and 710 μg [61], and was unknown in some studies.

The outcomes were both functional and histological. Functionally, the compound muscle action potential (CMAP), sciatic functional index (SFI) and gastrocnemius wet weight (WW) were mainly tested. Histologically, the cross-sectional area of the muscle fibers and the diameter of the axon were the main outcomes (Tables S1 and S2). These results were collected between 2 and 16 weeks with most results collected at 8 and 12 weeks.

### 3.2. Liu et al. [60]: UMSC-Derived Exosomes

Liu et al. investigated the effect of UMSC-derived exosomes (final concentration of 0.5 μg/μL) in vivo and in vitro in a hydrogel scaffold with different stiffnesses (100 μg/μL and 40 μg/μL) on the regeneration of a crush injury in the sciatic nerve (phosphate-buffered saline (PBS), a stiff hydrogel with and without exosomes, and a soft hydrogel with and without exosomes). Results were studied at 14 days’ post-injury in five groups and a sham group with no injury. They studied the release of exosomes in vivo and in vitro and found conflicting results. At 24 h in vitro, exosomes were released more rapidly by the stiff hydrogel than by the soft, whereas in vivo, the soft released them more rapidly. Concerning the regenerative effects at 14 days, the best statistically significant results were found in the soft hydrogel group, with the highest wet weight, larger surface and higher SFI. In conclusion, soft hydrogel with exosome showed a better regeneration and functional recovery.

### 3.3. Yang et al. [59] and Chen et al. [58]: ASC-Derived Exosomes

Two authors studied the effect of ASC-derived exosomes. Chen et al. evaluated the effect of human adipose stem cell (hASC)-derived exosomes (final concentration of 0.5 μg/μL) embedded in Matrigel poured into a silicone conduit on a 7 mm long sciatic nerve defect in vivo and in vitro. Matrigel and Matrigel with exosomes were compared. Yang et al. investigated the effect of rat ASC-derived exosome (final concentration of 0.5 μg/μL) supplemented with NFT3 in an alginate hydrogel/silicone scaffold on the regeneration of a 10 mm gap in the sciatic nerve in rats in vivo and in vitro.

Chen et al. assessed the in vitro uptake of exosomes by SCs and dorsal root ganglion (DRG) neurons and showed that it was much higher in SCs than DRGs at 2, 3 and 4 h. However, DRG neurites showed a significant outgrowth in the exosome solution compared to PBS only.

The effect of exosomes on SCs compared to incubation in PBS at 24, 48 and 72 h was denoted by an increased migration of SCs (with a dose and incubation time-dependent effect with a higher migration at concentrations of 20 μg /mL than 10 μg /mL) as well as an increase in mRNAs of neurotrophic factors (CNTF, NGF and BDNF) and genes responsible for SC proliferation and migration.

Yang et al. evaluated the in vitro exosome release rate of the alginate hydrogel into a PBS solution, which was at its highest in the first 4 days, by the end of which 80% of the exosomes were liberated into the PBS. The rate was then stable over the next 14 days. Yang et al. divided the in vivo experiments into four groups. They were made with the following scaffolds: silicone and alginate; silicone, alginate and exosomes (Exo); silicone, alginate and exosomes with NT3 (Exo-NT3) and autograft, respectively. Results were collected at 2 and 8 weeks, while Chen et al. gathered the in vivo results at 4 and 8 weeks.

Yang et al. found no difference in the functional recovery between the groups at 2 weeks. At 8 weeks, all groups had improved. The autograft group showed the most recovery, followed by the Exo-NT3; no statistical difference was observed between the Exo group and the alginate-only group. The CMAP was measured at 8 weeks and the highest amplitude and shortest latency were found in the autograft group, and were closely followed by the Exo-NT3 group. Again, no statistically significant difference was found between the alginate and Exo groups. The gastrocnemius wet weight, muscle fiber cross-section, and axon diameter showed the same pattern with the highest weight and cross-section in the autograft group and the lowest in the alginate and Exo group. The Exo NT3-enriched group promoted peripheral nerve regeneration, but there was no statistically significant difference between the Exo and non-Exo groups.

Chen et al. found that the gastrocnemius wet weight and muscle fiber cross-section area were significantly higher at 4 and 8 weeks in the exosome group compared to the PBS-only group. Regarding axonal regeneration, the number of myelinated axons and the myelin area were statistically higher in the exosome group. Histologically, the axons and myelin showed a denser arrangement. Exosome-enriched Matrigel promoted better peripheral nerve regeneration compared with non-exosome-enriched.

### 3.4. Ikumi et al. [64]: Purified Exosome Product (PEP)

Ikumi et al. investigated the effect of PEP in fibrin glue (Tisseel, Baxter, UK) poured in a reverse autograft on the regeneration of a 10 mm long sciatic defect in rats in vivo and in vitro. The in vivo regeneration was assessed in three groups: the reverse autograft group; the fibrin/autograft group; and the exosome fibrin/autograft group. At 12 and 16 weeks, the functional recovery was evaluated through the CMAP, the tibialis anterior wet weight, and isometric tetanic force, which measures the maximal force of the muscle. On these parameters, there was no statistically significant difference among the three groups, except for the isometric force, which was higher in the exosome group at 16 weeks. Regarding the histology of the post-regeneration sciatic nerve, the parameters examined were axon diameter and density. While there was no statistically significant difference in axon density between the groups, the exosome group showed a statistically significant larger axon diameter at 12 and 16 weeks. Markers of SC activation were evaluated in vitro, including S100b which is secreted by activated SCs and increases SC and Mac recruitment. RNA expression of s100b showed a four-fold increase compared to the autograft group. The authors concluded that PEP improved motor outcome and nerve regeneration and changed the gene profile.

### 3.5. Li et al. [61]: BMSC-Derived Exosomes

Li et al. investigated the effect of BMSC-derived exosomes in a polydopamine (PDA)-modified chitin scaffold on the regeneration of a 2 mm long sciatic nerve defect in rats in vivo and in vitro. In this model, the scaffolds were immersed in a 1 μg/μL solution of exosomes. A significantly higher concentration of exosomes was taken up by the modified PDA conduit. The release of exosomes was then studied in vitro and showed a faster release in the chitin-only group with an initial burst and little residue at 10 days versus a slower release with 20% of exosomes still present at 14 days in the modified PDA group. In vitro analysis of the mRNA expression of transcription factors (Jun and Sox2) and myelination genes (MBP and Krox20) showed a clear increase in Jun and Sox2 expression at days 1 and 5 in the Exo groups (greater at day 1 in the chitin group and greater at day 5 in the chitin/PDA group) and a decrease in MBP and Krox20 mRNA at 1 and 5 days.

For the in vivo experiment, they compared four groups at 8 weeks, chitosan and PDA-modified chitosan, without and with exosomes. For the functional recovery, the chitosan/PDA Exo group showed superior results to the other groups regarding SFI, wet weight and CMAP amplitude. Latency was similar to the chitosan Exo group, but superior to the groups without exosomes. The cross-section area of the muscle fibers was larger in the chitosan/PDA Exo group. Histologically, the density, diameter of the myelinated axons and thickness of the myelin sheet were statistically higher in the Exo groups, particularly in the chitosan/PDA group. In conclusion, the chitosan/PDA Exo group was more efficient in peripheral nerve regeneration than its chitosan counterpart.

### 3.6. Rao et al. [62]: GMSC-Derived Exosomes

Rao et al. examined the effect of GMSC-derived exosomes (concentration 1 μg/μL) into a chitin conduit on the regeneration of a 10 mm long sciatic defect in rats in vivo and in vitro.

In vitro, the effect of exosomes on SCs and DRG neurites was observed, demonstrating a greater proliferation of SCs at 5 days in the exosome group versus the non-exosome group and a statistically significant growth of neurites. In vivo experiments were conducted in three groups: autograft, chitin, and chitin and exosomes (Exo group). Functional recovery was assessed at 4, 8 and 12 weeks for the SFI and 12 for the wet weight and CMAP. There was no difference between the groups at 4, 8 and 12 weeks. The autograft group showed the best results; the Exo group presented statistically better results than the chitin group, but at 12 weeks, the results were similar to those of the autograft. The wet weight was statistically higher in the Exo group at 12 weeks and similar to that of the autograft. Concerning electrophysiology, CMAP latency was statistically shorter in the Exo group and the amplitude was higher than in the chitin group; the autograft had the best results. Not surprisingly, the gastrocnemius’s fiber cross-section was considerably larger in the Exo group and similar to the autograft group. The same pattern was found in the histological analysis of the nerves at 12 weeks with a significant improvement in the number of myelinated fibers, their diameter and the thickness of the myelin sheath in the exosome group, but a better outcome was observed in the autograft group. The authors concluded that exosomes provided function, axon regeneration, myelinization, nerve conduction and a higher diameter with results similar to autografts in some cases.

### 3.7. Pan et al. [63]: Induced Human Pluripotent Stem Cell (iPCS)-Derived Exosomes

Pan et al. studied the effect of iPCS-derived exosome in an ANG on the regeneration of a 15 mm long sciatic nerve defect in rats in vivo. The in vivo experiment comprised three groups (an exosome/acellular graft group, an acellular group and an autograft group) compared at 1, 2, 4, 8 and 12 weeks. Functional recovery was tested through the SFI, the gastrocnemius wet weight, the isometric force and the CMAP. At weeks 1, 2 and 4, there was no difference between the three groups regarding SFI and wet weight, although the latter decreased over the first 4 weeks. The CMAP was undetectable at weeks 1 and 2 and only weakly detectable at week 4. By week 8, a statistically significant difference appeared between the Exo group and the acellular graft group for SFI and wet weight. Regarding electrophysiology, the latency of the CMAP was notably lower in the exosome and autograft groups. For all the previously mentioned measures, the best results were found in the autograft group. Histologically, the cross-section of muscle fibers showed a difference at 4 weeks with a statistically significant difference in the autograft and exosome groups with larger fibers. Nerve histology was assessed at 8 and 12 weeks. Consistent with the other results, the number of axons, their surface area and diameter were greater in the exosome and autograft groups. Interestingly, the greatest number of axons was found in the exosome group, which was statistically significantly higher than the autograft group. The result in the exosome group showed that motor function, axon diameter, and muscle reinnervation were comparable to autografts.

## 4. Discussion

In this review, we compared seven original studies on the effects of nerve conduits mounted with exosomes on peripheral nerve regeneration. Overall, promising results are reported with a significant improvement in function and histological parameters. Apart from the study by Liu et al. [60], no study was performed using different conduits or conduit stiffnesses. Of note, stiffer conduits showed better exosome release, but softer conduits had better functional outcomes. The process of exosome production involves several steps. Initially, early endosomes are formed by an intracellular budding of the cell’s plasma membrane. Intraluminal vesicles will then form, with the help of the Golgi apparatus, and thus transform the early endosome into a late endosome (or multivesicular body). Cargo molecules such as RNA, proteins and lipids will then be added into these intraluminal vesicles. Eventually, some multivesicular bodies will fuse with the cell’s plasmatic membrane and release exosomes, identifiable by the presence of tetraspanins such as CD81 and CD63 on their membrane [47], into the extracellular space [50,65]. These exosomes contain DNAs, messenger RNAs, miRNAs, cytokines and proteins [66], whose RNA classes depend on the role of the secreting cell.

In the field of peripheral nerve regeneration, exosomes have been studied in vivo and in vitro in their interaction with SCs, neurons and macrophages. Concerning SCs, exosomes are internalized in a dose-dependent manner. In vitro, exosomes promote SC proliferation by activating cyclin genes as well as migration. They also appear to upregulate the expression of neurotrophic factors from SC, such as BDNF, CNTF and NGF [53,58,65]. Numerous NTFs have also been identified in exosomes, as well as factors required for myelin phagocytosis (galectin 3) and remyelination (MAG and PLP) [53,65]. In vivo, a greater density of the myelin sheath, as well as an increase in the number of axons (at 1 and 2 months) and a better reinnervation of the muscle have been demonstrated following the use of ASC-derived exosomes. Parameters such as muscle wet weight, cross-section of the muscle fibers, the SFI and the CMAP are used to evaluate the reinnervation of the muscle and the functional outcome [58,64]. As to the interaction of exosomes with neurites, exosomes can be internalized and transported in a retrograde fashion to the body of the neuron in vitro and in vivo [67]. Studies reported exosomes to promote axonal growth and to increase neurite length [53]. This has an important impact when considering the risks of chronic denervation at the distal stump. MSC exosomes also have an immunomodulatory role through the induction of anti-inflammatory cytokines such as IL10 and TGFβ, and the downregulation of inflammatory factors like TNFα and IL1β, and through the polarization of macrophages towards an M2 phenotype—the anti-inflammatory type [49]. MSC-derived exosomes also have a role in vascular regeneration, studied mostly in the central nervous system where exosomes have been shown to increase the recruitment of endothelial cells leading to better revascularization [51]. Such an effect on all the actors of peripheral nerve regeneration offers great perspectives.

One of the mechanisms by which exosomes act is through their content in miRNA, a type of non-coding RNA that plays a post-transcriptional role in the regulation of mRNAs of the recipient cell by binding to them and blocking their transcription or breakdown [49,65]. MiRNAs seem to play an important role in the exosome–neurite interaction, as studies where miRNA expression is limited (through Argo2 knockout) showed a decrease in neurite elongation [68].

## 5. Challenges in the Field and Future Directions

The exosome origin was diverse in the studies included in our review (PEP, ASC, MSC, UMSC, BMSC and iPSC). Most of these sources, except for PEP, require de-differentiation steps to reach the stem cell stage, allowing for the production of exosomes. Mesenchymal stem cells (MSCs) represent another promising source, as they can be isolated from bone marrow (BMSCs), adipose tissue (ASCs), and the umbilical cord (hUC-MSCs). Their immunomodulatory effects make them particularly interesting, and depending on their origin, they are easier to extract, especially ASCs. However, as MSCs are transferred, they proportionally lose their capacity to produce exosomes [69,70]. Induced pluripotent stem cells (iPSCs) are attractive given their origin as de-differentiated somatic cells. However, their de-differentiation into stem cells may be incomplete or lead to greater cellular diversity compared to natural stem cells, with the risk of poorly de-differentiated cells [71]. PEP is a lyophilized exosome preparation that needs to be diluted before use, enabling exosomes to be used relatively quickly and conveniently. Sources can be donor plasma, which requires filtration and centrifugation to be isolated [64,72,73].

Among these exosome sources, the most applicable to the clinical setting appear to be ASCs and iPSCs for their ease of extraction and their abundance, and PEPs, which do not require preparation, are easy to store and come from an accessible source. The same variability was observed in the scaffolds used: hydrogel, chitin, and chitosan (+/−PDA); autograft and fibrin; ANG; alginate; and Matrigel poured into silicone fillings with gaps from 15 mm to simple crush injuries. The exosome concentration was highly variable and unknown for some studies [63,64], ranging from 0.5 μg/μL to 1 μg/μL with total concentrations from 10 μg to 710 μg.

Given the wide range of data and the limited number of studies on the subject, it is difficult to make an exact comparison of the data. However, we have identified some general trends.

Apart from the study comparing simple exosomes with those enriched with NT3 where there was no significant difference between exosome and exosome-free groups, a significant difference was found in the outcomes when exosomes were added to the different scaffolds.

Histologically, nerve maturation can be observed through axon diameter, the presence and thickness of the myelin sheath, and the regularity of nerve fibers. An increase in diameter may suggest accelerated nerve maturation [64]. The number of axons may reflect the trophic effect induced by exosomes through both anti-apoptotic and proliferative actions on axons [63]. In this study, the diameter of axons and the muscle cross-section area were compared at 8 and 12 weeks in several studies. In both regards, the ANG scaffold showed the best results at 8 and 12 weeks, with an axonal diameter almost eight times larger at 8 weeks than the alginate matrix and silicone tube (1.1 μm without the addition of NT3 and 1.4 μm with NT3). However, the same matrix showed a muscle cross-sectional area of 1100 μm at 8 weeks (for NT3-enriched exosomes). In addition, the ANG scaffold with exosomes showed a diameter of muscle fibers and a muscle fiber area similar to that of autograft. The fibrin matrix in a reverse nerve graft diameter was 3.16 μm at 12 weeks, almost three times smaller than the ANG matrix.

The wet weight represents the weight of the muscle (gastrocnemius mostly or tibialis anterior) at the time of its extraction postoperatively, after being drained of its blood. It is correlated to muscle atrophy following denervation. The operated side is compared to the healthy side and the results are usually given as a percentage of the healthy side, whereby the higher the wet weight, the better the reinnervation [58]. The chitin duct showed the best results at 12 weeks with a wet weight of 77% on the injured side, followed by the ANG duct at 65%. At 8 weeks, the best results were found in the chitosan–PDA conduit with 70% of wet weight, but it is necessary to consider the nerve gap of only 2 mm for this conduit. The sciatic functional index (SFI) is designed to evaluate the functional recovery after a sciatic nerve injury. It is based on three parameters: the length of the step, the distance between the toes (1 to 5), and the intermediate distance between the toes (2 to 4), which are measured on a paper track. The results are given on a scale of 0 to −100, where the sciatic function is normal at 0 and completely absent at −100 [74]. It evaluates the proper connection between the motor axon and the motor endplate [63]. The neuromuscular function illustrated by the SFI showed similar results, with the best results for the chitosan–PDA conduit (−50) at 8 weeks. The chitin and ANG conduits performed less with an SFI at −75, but demonstrated a noticeable improvement with results of around −60 at 12 weeks.

Compound muscle action potential (CMAP) is an electrophysiological test assessing sciatic nerve conduction latency and amplitude using electrodes placed at the paraspinal sciatic origin and a receiving pair in the gastrocnemius. A current (0.09 mA for 0.1 ms) is applied and CMAP recorded. Latency inversely relates to myelin thickness, while amplitude correlates with fiber count [59,62]. Interestingly, although the ANG conduit showed the best histological results with the largest axonal diameter when one considers its CMAP, the latency was the greatest at 8 weeks (3 ms) with little improvement at 12 weeks (2.7 ms). However, the nerve gap bridged in this study was the largest at 15 mm, which might also have an impact on the results. The lowest latency (2 ms) was found in the chitin duct with an amplitude of 9 ms at 12 weeks, which would indicate the best myelinization. The same studies showed the best SFI (−60) at 12 weeks, immediately followed by the ANG (−62) group.

The gap sizes vary significantly across studies, ranging from crush to 15 mm. This variation must be considered when analyzing the results, as it complicates direct comparisons between datasets. Notably, Li et al. mainly reported the best outcomes at eight weeks with a 2 mm gap. The risk of PNR failure is lower with smaller gaps compared to larger ones (15 mm in the study), especially considering that the critical nerve gap in rats is reported to be 1.5 cm, which corresponds to the critical 4 cm gaps in human [1]. Even though one cannot directly extrapolate the findings to humans, the study using a rat model provides good insights for the further developments of scaffolds towards clinical use.

One of the main questions of this review was to identify the ideal scaffold for exosome delivery to peripheral nerves. Key known characteristics are porosity and permeability [14], which allow for the diffusion of nutrients and oxygen without letting other cells infiltrate the duct. Studies state that the ideal pore size is between 1 and 40 μm with a total duct porosity of 80% [17,75].

Silicone, the oldest artificial conduit used, has many shortcomings, inducing chronic inflammatory reactions whose fibrosis may compress the newly formed nerve and requiring sometimes a second operation to be removed [75]. Synthetic polymers including PLA (poly-lactic acid), PLC (poly-ε-caprolactone) and PGA (polyglycolic acid) offer advantages such as their high mechanical strength and adjustable degradation time according to their composition and cross-linking. They are also less immunogenic than conduits from allografts [76].

Natural polymers include autologous non-neuronal conduits and decellularized allo/xenografts, as well as extracellular matrix derivatives (collagen, laminin, fibrin, fibronectin), polysaccharides (chitosan, alginate, agarose) and proteins (silk fibroin, keratin) [14]. Natural polymers present advantages, such as their biocompatibility and adhesive capacity (especially molecules derived from extracellular matrix), although their mechanical strength is limited. Alginate is biodegradable and well tolerated, but requires calcium binding to form a solid hydrogel, which can be cytotoxic in large quantities. Chitin has high porosity, biodegradability and a predictable degradation rate, maintaining structural integrity for at least six weeks [62]. Additionally, it offers non-toxicity, biocompatibility and water permeability. However, it exhibits low mechanical strength, which may limit its direct applications [77]. Chitosan, the deacetylated form of chitin, interacts with negatively charged neurons due to its positively charged surface and has been shown in some studies to enhance nerve regeneration. However, it has limited mechanical strength in physiological conditions and needs to be cross-linked with collagen or gelatin [78]. They may also present an immunological risk if they are derived from animals and a significant cost if they are created in a laboratory [78]. Scaffolds such as fibrin have been shown to promote angiogenesis and, compared to microsuture alone, promotes better fiber alignment, better axonal regeneration and reduces inflammation [7,8,9]. However, the low tensile strength of natural polymers such as fibrin [15] requires the scaffold to either be microsutured [79,80] or put into a stronger conduit [81] as it has a high risk of dehiscence. To improve their characteristics, natural polymers can be combined, together or with synthetic polymers [18]. However, their density might impede cell colonization [14,18]. Recent studies are investigating the potential to enhance scaffold qualities by incorporating molecules such as fisetin, known for its immunomodulatory properties [82], or focusing on the thermosensitivity of hydrogels to ensure a slower release of extracellular vesicles [83]. Allografts, made from decellularized nerve tissue, veins, arteries, tendons or muscles, can be used to fill gaps. Nerve allografts are decellularized conduits processed through various chemical methods and then sterilized. They retain the native 3D internal structure of the nerve, making them particularly advantageous conduits. However, their clinical use is limited by the limited number of donor sources and the requirement for subfreezing storage conditions. Despite these limitations, nerve allografts have been shown to be superior to collagen conduits in repairing nerve gaps greater than 15 mm in digital nerves [84]. Veins can fill gaps up to 3 cm, or longer gaps if collagen is added, which can cause venous fibrosis. The use of arteries is debated due to inconsistent results. Muscle fibers have been evaluated, but neuromas can form as nerve fibers grow on the outside [18]. Not examined in the other studies, but investigated by Liu et al., is the importance of the scaffold stiffness. In their study, soft hydrogel with exosomes showed a significant improvement in the SFI (−47.88 vs. 79.36) as well as wet weight (61.63 vs. 45.85) compared with the stiff hydrogel through a quicker release of exosomes in the soft group and modulation of the inflammatory response.

Research is focusing on new, more effective scaffolds to overcome the burst release of exosomes. One example is a Novel Superparamagnetic Multifunctional Nerve Scaffold with SCs, which explores inducing exosome secretion directly in the patient through a rotating magnetic field that exerts mechanical force on SCs, promoting long-term exosome production [85]. Research also focuses on preconditioning stem cells—culturally, biochemically, mechanically, or environmentally. Exosome content depends on both the parent cell and the environment in which they are secreted. It has been shown that culture in a hypoxic environment favors the pro-angiogenic effect of exomes, while 3D culture, as opposed to traditional 2D culture, optimizes their function and paracrine activity [86]. Similarly, the stimulation of MSCs by pro-inflammatory cytokines shows an immunomodulatory effect of exosomes [87]. Finally, mechanical stimulation, for example, via a vertical-wheel bioreactor, can increase exosome yield nearly sixfold via shear stress on MSC plasma membranes [86].

## 6. Conclusions

Chitin and ANG scaffolds appear to be the most promising scaffolds, both histologically and functionally. Among the exosome sources, the most applicable to the clinic appear to be ASCs and iPSCs for their ease of extraction and their abundance, and PEPs, which do not require preparation. However, exosomes clinically applied to peripheral nerve regeneration is still a relatively new technique. More studies with the same origin and exact concentrations of exosomes used comparing gaps of a similar size, as well as a comparison of different scaffolds’ stiffnesses and long-term results, are needed in order to draw better conclusions.

## Figures and Tables

**Table 1:** Summary of the selected articles, stem cell origin, scaffolds, gap size and outcomes.

Exosomes	Scaffold	Study Model	Defect	Outcome	Author	Year	Reference
UMSC [0.5 μg/μL]	Hydrogel (HAMA) (stiff: 100 μg/μL; soft: 40 μg/μL)	in vivo and in vitro rat sciatic nerve	Crush injury	The stiffness of the hydrogel changed the dynamic of exosome release. Soft hydrogel showed better PNR and functional recovery.	Liu et al.	2022	[60]
hASC [0.5 μg/μL]	Matrigel in a silicone scaffold	in vivo and in vitro rat sciatic nerve	7 mm	ASC Exo promoted PNR via SC upregulation, with better myelinization and axonal regeneration.	Chen et al.	2019	[58]
ASC (+NT3) [0.5 μg/μL]	Alginate hydrogel in a silicone scaffold	in vivo and in vitro rat sciatic nerve	10 mm	Exo NT3 improved the PNR, with no difference between the Exo and non-Exo groups.	Yang et al.	2021	[59]
PEP (5%)	Reverse autograft with fibrin glue	in vivo and in vitro rat sciatic nerve	10 mm	PEP improved motor outcome and nerve regeneration and changed the gene profile.	Ikumi et al.	2021	[64]
BMSC Chitosan 364 μg	Chitosan (+/− PDA modified)	in vivo and in vitro rat sciatic nerve	2 mm	The chitosan PDA Exo group was more efficient than its chitosan counterpart on PNR; chistosan PDA Exo released Exo in a slower pattern.	Li et al.	2022	[61]
GMSC [1 μg/μL]	Chitin	in vivo and in vitro rat sciatic nerve	10 mm	Exosomes provided better motor function, axon regeneration, myelinization and nerve conduction as well as higher diameter. Autografts provided better results.	Rao et al.	2019	[62]
iPSC	Acellular nerve grafts (ANGs)	in vivo rat sciatic nerve	15 mm	Motor function, axon diameter and muscle reinnervation were comparable to autografts.	Pan et al.	2022	[63]

## Data Availability

No new data were created or analyzed in this study. Data sharing is not applicable to this article. All information relevant to this systematic review is part of the manuscript, figures, tables, and/or digital supplemental content. Additional information can be found within the publicly available PROSPERO protocol for this study. If any further information is required, the reader may contact the corresponding author for clarifications.

## Supplementary Materials

The following supporting information can be downloaded at: https://www.mdpi.com/article/10.3390/ijms25126489/s1.
