# Supplementary material for: Therapeutic Potential and Challenges of Mesenchymal Stem Cell-Derived Exosomes for Peripheral Nerve Regeneration: A Systematic Review
Source: Int J Mol Sci. 2024 Jun 12;25(12):6489. doi: 10.3390/ijms25126489 (PMC11203969; doi:10.3390/ijms25126489)
Supplement: Supplementary file 1 [file ijms-25-06489-s001.zip › ijms-3022759-supplementary.pdf]

Table S1. Histological outcomes at 8 and 12 weeks of exosome-enriched scaffolds.

	Weeks	Result	Scaffold	Author
**Axon diameter**	8	1.1 µm (wo NT3)<br>1.4 µm (+ NT3)	Alginate gel within a silicone tube	Yang et al.
		4.5 µm<br>5.5 µm	Chitosan<br>Chitosan + PDA	Li et al.
		8 µm	ANG	Pan et al.
	12	3.16 µm	Fibrin within a reversed autograft	Ikumi et al.
		6.2 µm	Chitin	Rao et al.
		9µm	ANG	Pan et al.
**Muscle cross-section area**	8	500 µm3	Matrigel within a silicone tube	Chen et al.
		550 µm3<br>650 µm3	Chitosan<br>Chitosan +PDA	Li et al.
		850 µm3 (wo NT3)<br>1100 µm3 (+ NT3)	Alginate gel within a silicone tube	Yang et al.
		1000 µm3	ANG	Pan et al.
	12	1400 µm3	Chitin	Rao et al.
		1500 µm3	ANG	Pan et al.

Table S2. Functional outcomes at 8 and 12 weeks of exosome-enriched scaffolds.

	Weeks	Result		Scaffold	Author
**Wet weight**	8	40%		Matrigel within a silicone tube	Chen et al.
		48%		ANG	Pan et al.
		50%<br>70%		Chitosan<br>Chitosan + PDA	Li et al.
	12	57% (TA)		Fibrin within a reversed autograft	Ikumi et al.
		65%		ANG	Pan et al.
		77%		Chitin	Rao et al.
**SFI**	8	-75		Chitin	Rao et al.
		-75		ANG	Pan et al.
		-71 (wo NT3)<br>-62 (+ NT3)		Alginate gel within a silicone tube	Yang et al.
		-60<br>-50		Chitosan<br>Chitosan + PDA	Li et al.
	12	-62		ANG	Pan et al.
		-60		Chitin	Rao et al.
**CMAP**	8	*Latency*	*Amplitude*		
		3ms		ANG	Pan et al.
		3ms<br>2.5ms	7mv (wo NT3)<br>13mv (+ NT3)	Alginate gel within a silicone tube	Yang et al.
		2.5ms<br>2.2ms	11mv<br>14mv	Chitosan<br>Chitosan + PDA	Li et al.
	12	2.7ms		ANG	Pan et al.
		2ms	9mv	Chitin	Rao et al.
